# Tissue-Specific Transcriptomics of the Exotic Invasive Insect Pest Emerald Ash Borer (*Agrilus planipennis*)

**DOI:** 10.1371/journal.pone.0013708

**Published:** 2010-10-28

**Authors:** Omprakash Mittapalli, Xiaodong Bai, Praveen Mamidala, Swapna Priya Rajarapu, Pierluigi Bonello, Daniel A. Herms

**Affiliations:** 1 Department of Entomology, Ohio Agricultural and Research Development Center, The Ohio State University, Wooster, Ohio, United States of America; 2 Department of Plant Pathology, The Ohio State University, Columbus, Ohio, United States of America; Yale School of Medicine, United States of America

## Abstract

**Background:**

The insect midgut and fat body represent major tissue interfaces that deal with several important physiological functions including digestion, detoxification and immune response. The emerald ash borer (*Agrilus planipennis*), is an exotic invasive insect pest that has killed millions of ash trees (*Fraxinus* spp.) primarily in the Midwestern United States and Ontario, Canada. However, despite its high impact status little knowledge exists for *A. planipennis* at the molecular level.

**Methodology and Principal Findings:**

Newer-generation Roche-454 pyrosequencing was used to obtain 126,185 reads for the midgut and 240,848 reads for the fat body, which were assembled into 25,173 and 37,661 high quality expressed sequence tags (ESTs) for the midgut and the fat body of *A. planipennis* larvae, respectively. Among these ESTs, 36% of the midgut and 38% of the fat body sequences showed similarity to proteins in the GenBank nr database. A high number of the midgut sequences contained chitin-binding peritrophin (248)and trypsin (98) domains; while the fat body sequences showed high occurrence of cytochrome P450s (85) and protein kinase (123) domains. Further, the midgut transcriptome of *A. planipennis* revealed putative microbial transcripts encoding for cell-wall degrading enzymes such as polygalacturonases and endoglucanases. A significant number of SNPs (137 in midgut and 347 in fat body) and microsatellite loci (317 in midgut and 571 in fat body) were predicted in the *A. planipennis* transcripts. An initial assessment of cytochrome P450s belonging to various CYP clades revealed distinct expression patterns at the tissue level.

**Conclusions and Significance:**

To our knowledge this study is one of the first to illuminate tissue-specific gene expression in an invasive insect of high ecological and economic consequence. These findings will lay the foundation for future gene expression and functional studies in *A. planipennis*.

## Introduction

The insect midgut and fat body are important metabolic tissues and represent key physiological interfaces during interactions with hosts. While both tissues are highly specialized and play major roles in the life of insects, they possess discrete physiological functions essential for growth and development. The midgut plays important roles in digestion, antioxidant defense and is thought to be one of the primary interfaces in countering the effects of dietary toxins [1]–[5], xenobiotics (ex. insecticides) and potential pathogens [6]. On the other hand, the insect fat body is a dynamic tissue, which plays vital roles in intermediary metabolism, energy storage/utilization, detoxification and immune response [7], [8]. Remarkably, functions performed by the insect fat body are correlated to those of adipose tissue and liver of vertebrates. The combined action of the factors associated with the midgut and fat body could determine the capacity of an insect to adapt to various ecological niches and thereby evolve.

Beetles are the most predominant group of insects constituting about 25% of all known life-forms [9]. Within Coleoptera, the Buprestidae, known commonly as metallic wood-borers, remains one of the most understudied groups of insects despite their ecological importance in forests and the status of some species as key pests of trees. For example, emerald ash borer (*Agrilus planipennis* Fairmaire) which is endemic to Asia, has emerged as a devastating pest of ash trees in North America where it continues to kill millions of ash trees, primarily in the Midwestern United States and Ontario, Canada [10], [11]. All North American ash that it has encountered, including green ash (*Fraxinus pennsylvanica* Marshall), white ash (*F. americana* L.) and black ash (*F. nigra* Marshall) are highly susceptible [11]–[13]. If *A. planipennis* sustains its current rate of invasion, it is considered to have the potential to decimate ash on a continental scale [14], [15] with economic and ecological impacts reminiscent of the invasions of the chestnut blight and Dutch elm disease pathogens [16], [17].

Larvae of *A. planipennis* feed on the phloem and cambial tissues of ash trees, which usually results in the formation of S-shaped galleries that disrupt nutrient and water translocation, generally killing the trees in 3–5 years following attack [10]. *A. planipennis* is endemic to Far East Asia, including China, Korea, Japan and Siberia [18]. However, reports indicate that *A. planipennis* is rarely considered a pest in Asia [19], [20], where Manchurian ash (*F. mandshurica* Rupr) is a primary host [21], [22], and infestations appear restricted to stressed trees [23]. This implies that Manchurian ash may be generally resistant, with weakened trees preferentially colonized. A common garden experiment in Michigan confirmed that Manchurian ash has a much higher level of resistance to *A. planipennis* than do common North American ash species [24] and it has been reported that the resistance may be mediated by phenolic compounds in the phloem such as hydroxycoumarins and phenylethanoids, among other possible mechanisms [25].

Despite its status as a key pest, no functional genomics data exist for *A. planipennis*. In general, little is known at the tissue- level about the physiology-driven molecular strategies that invasive insects deploy to adapt to their new environment. This lack of fundamental molecular knowledge hinders our understanding of the insect's biology. However, the advent of second-generation sequencing such as 454 pyrosequencing [26] and Illumina/Solexa [27] offers a unique opportunity to study functional genomics [28] in non-model organisms. For example, the 454 pyrosequencing strategy has been successfully applied to unravel transcriptomic signatures in the tobacco hornworm, *Manduca sexta* L. [3], the soybean aphid, *Aphis glycines* [29] and the poplar leaf beetle, *Chrysomela tremulae* F. [30]. Here, we present data derived from 454 pyrosequencing of the midgut and fat body of *A. planipennis* larvae. Results obtained from this study will provide the basis for performing future gene expression and functional studies in *A. planipennis*. Furthermore, the data will improve our understanding of the biology of this key invasive pest at the molecular level.

## Results and Discussion

### Transcriptome analysis

We obtained 126,185 transcriptomic reads for the midgut and 240,848 reads for the fat body of third-instar *A. planipennis* larvae. The reads for both tissues were assembled using Newbler program (Roche) after the removal of adapter sequences. To achieve better consistency, the contigs and singletons of the transcripts were renamed in the format “EABMG000001” with “EAB” for emerald ash borer, “MG” for midgut, and “000001” for an arbitrarily assigned number. Similar nomenclature was adopted to describe the fat body transcriptomic sequences with MG replaced by FB for fat body.

**Figure 1:**
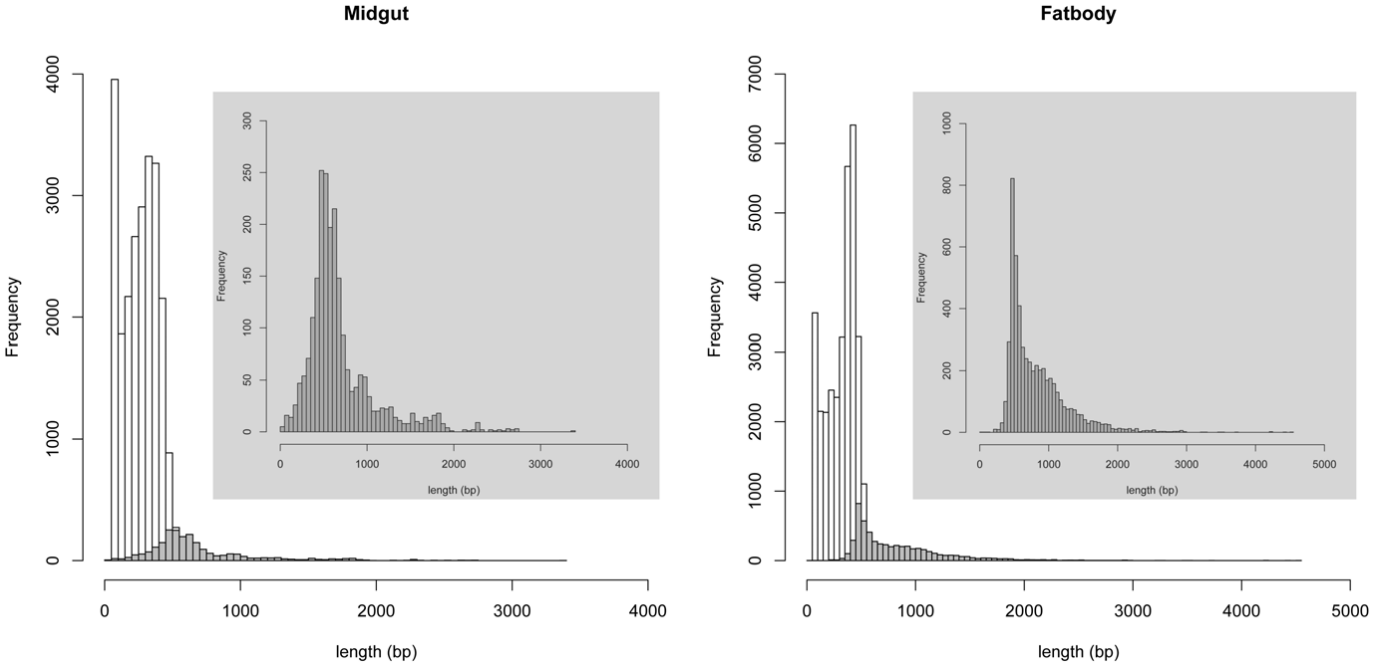
Summary of transcriptomic sequences from *Agrilus planipennis* midgut and fat body. The singleton sequences are represented by clear bars and the contig sequences by shaded bars (inserts).

After the assembly of the sequences, 25,173 high quality ESTs (2,218 contigs and 23,495 singletons) were obtained for the midgut and 37,661 ESTs (5,376 contigs and 32,285 singletons) for the fat body (Table 1). The singleton sequences for the midgut ranged from 50 bp to 710 bp with an average length of 259 bp and total length of 6,091,300 bp; while the contig sequences ranged from 36 bp to 3,395 bp with an average length of 688 bp and total length of 1,526,781 bp. The singleton sequences for the fat body ranged from 50 bp to 606 bp with an average length of 314; while the contiguous sequences ranged from 38 bp to 4,501 bp with an average length of 825 bp and total length of 4,433,073 bp (Figure 1).

**Table 1:** Summary of BLASTx search of the *Agrilus planipennis* sequences.

	MG*	FB
*Significant matches*	9,066	14,253
Artificial sequences	3	4
Archaea	1	0
Arthropoda	8,574	13,438
Bacteria	37	50
Other eukaryotes	448	757
Viruses	3	4
*Non- significant matches*	16,107	23,408
Total	25,173	37,661

* MG  =  midgut; FB  =  fat body.

Among the midgut transcripts, 9,066 (36%) showed significant similarity (E value < 1e−^5^ ) to proteins in the GenBank nr database, while 14,253 (38%) sequences of the fat body ESTs showed significant hits (Table 1). The majority of the transcripts with similar sequences in the database (95%) matched to arthropodal proteins. The remaining midgut and fat body transcripts were similar to proteins of non-insect eukaryotes (5%) and bacteria (0.02%). A total of 14 sequences were similar to viral proteins and artificial sequences; and one midgut sequence matched to an *Archaea* protein (Table 1).

### Comparative analysis

**Figure 2:**
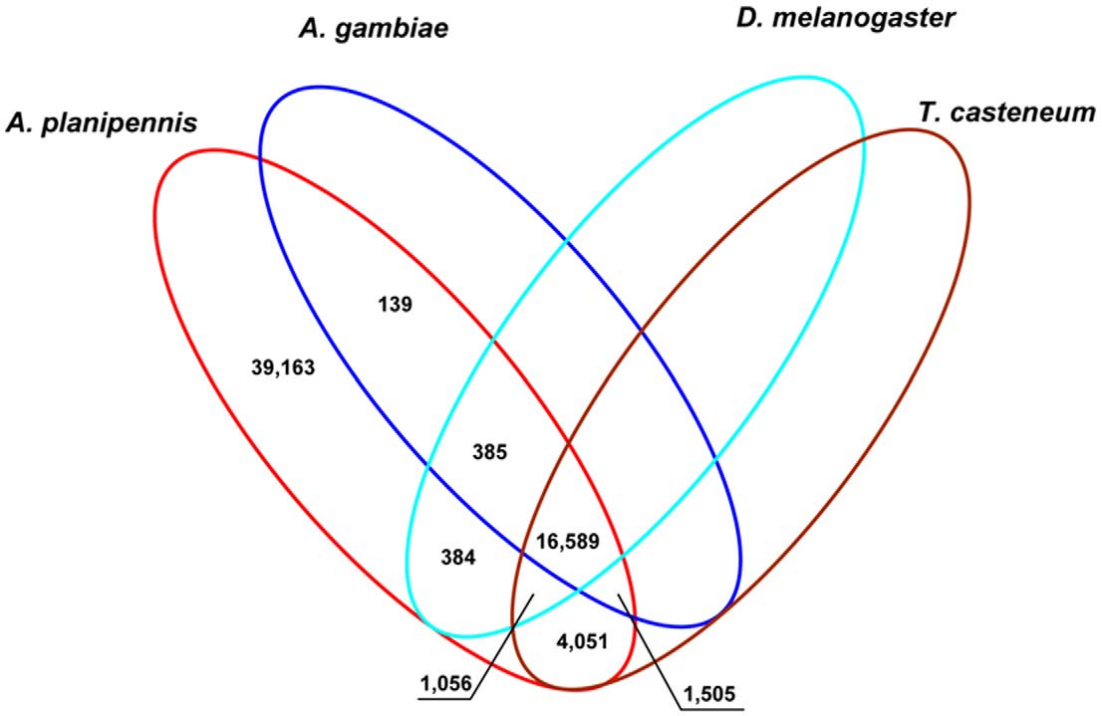
Summary of the comparisons of *Agrilus planipennis* transcriptomic sequences with protein sequences from the draft genomes of *Anopheles gambiae*, *Drosophila melanogaster*, and *Tribolium casteneum*.

A comparative analysis of the derived *A. planipennis* midgut and fat body transcripts with protein sequences in the draft genomes of *Drosophila melanogaster* Meigen, *Anopheles gambiae* Giles, and *Tribolium casteneum* Hebst, revealed most sequence similarity (36.6%, 23,201 out of 62,834) to the *T. casteneum* genome [31] (Figure 2). Similar observations were reported by Pauchet et al. (2009) [30] for the midgut transcriptome of the chrysomelid *C. tremulae*. Nearly equal proportions of *A. planipennis* sequences showed hits to the non-beetle genomes including *A. gambiae* (29.4%) and *D. melanogaster* (29.1%; Figure 2). A total of 16,589 sequences were shared among all four insect species under comparison and about 62% of sequences (39,228 out of 62,834) did not show BLASTX similarity, implying either that they represented untranslated regions, nonconserved coding regions, or genuinely novel proteins of *A. planipennis* (Figure 2 and Supplemental Table S1).

### Gene Ontology

**Figure 3:**
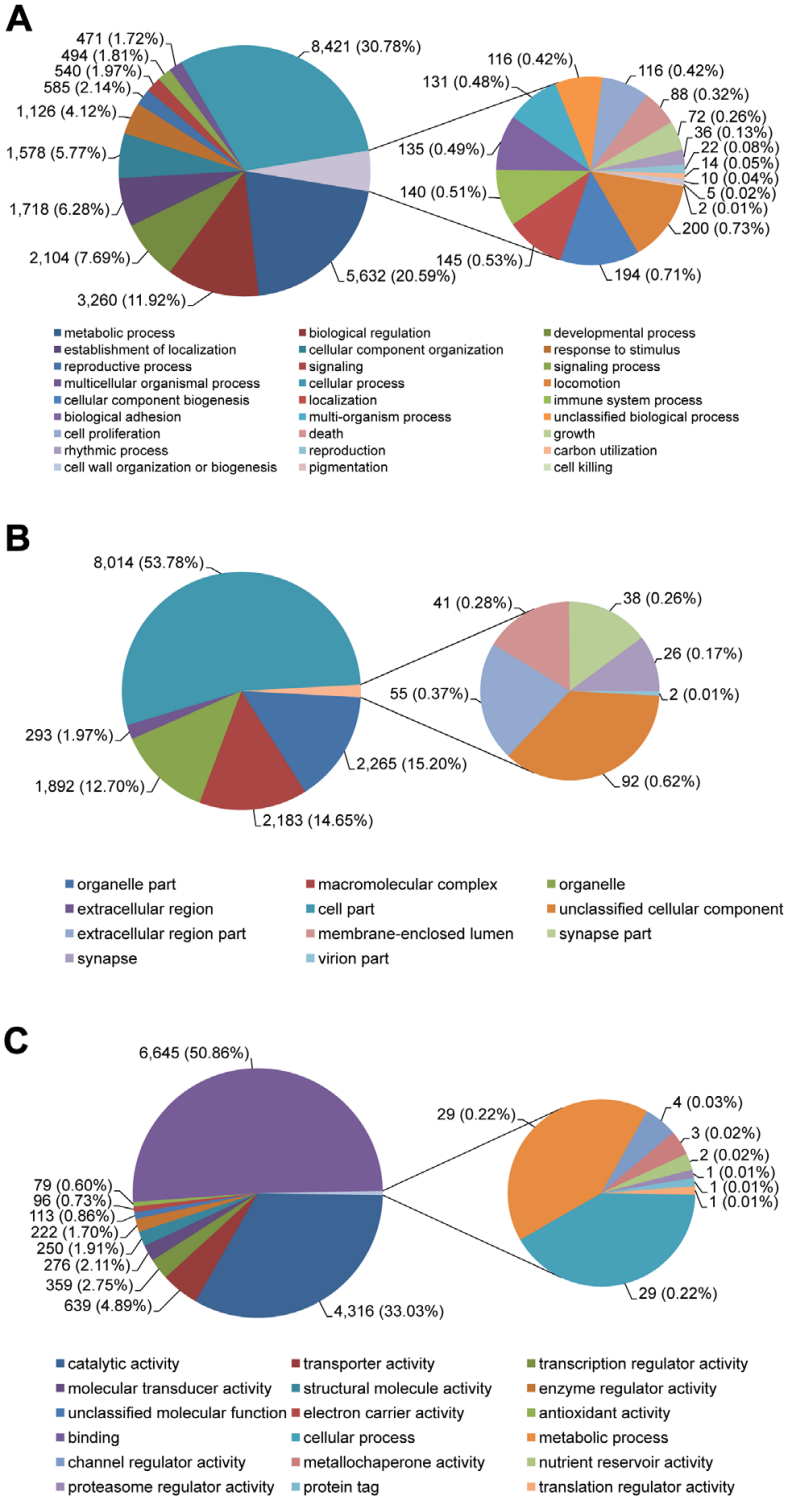
Gene ontology (GO) terms for *Agrilus planipennis* larval midgut transcriptome. (A) Biological Process, (B) Cellular Component and (C) Molecular Function.

**Figure 4:**
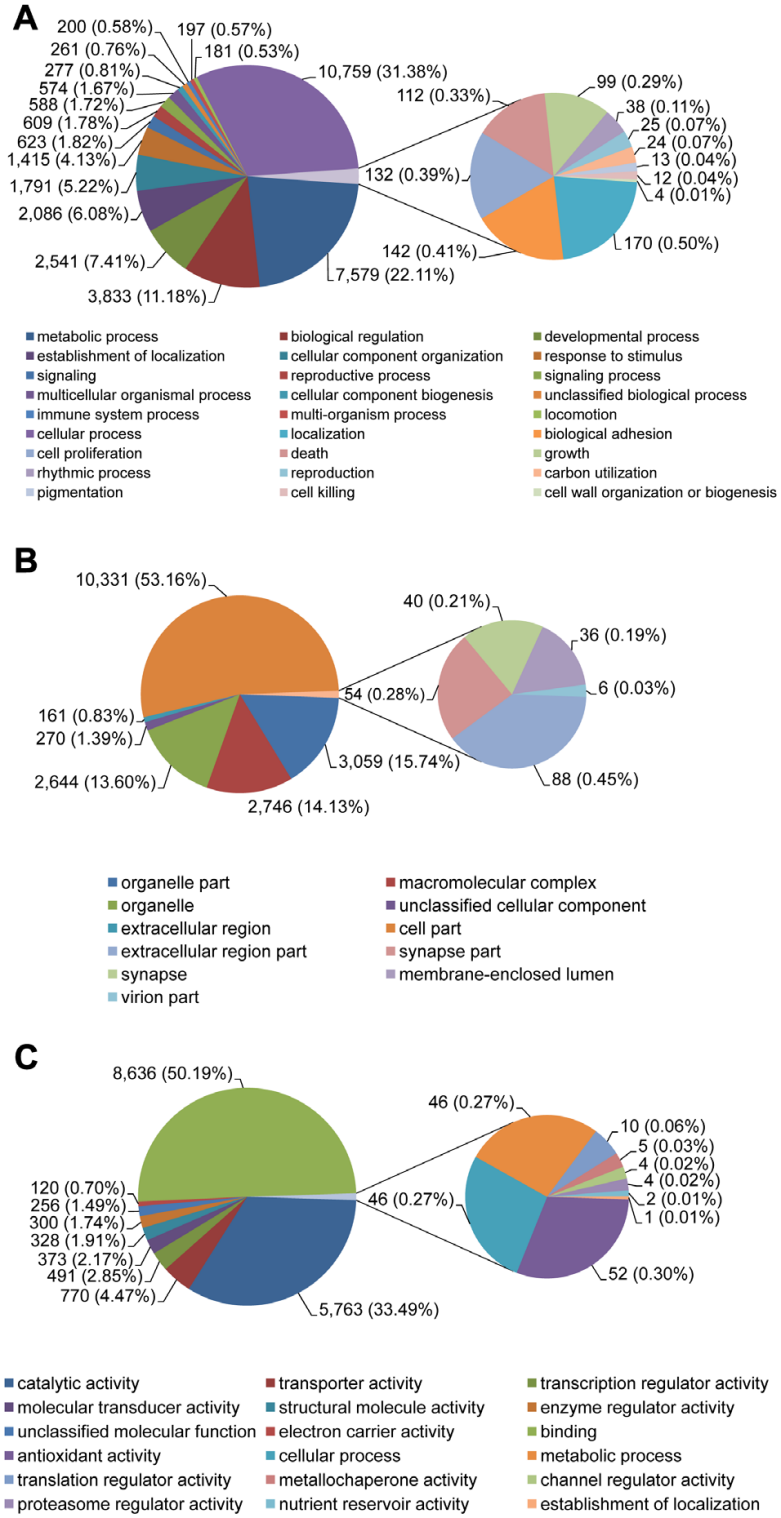
Gene ontology (GO) terms for *Agrilus planipennis* larval fat body transcriptome. (A) Biological Process, (B) Cellular Component and (C) Molecular Function.

The obtained unigenes were assigned Gene Ontology (GO) based on significant homologies to proteins in GenBank. Since the two EST databases represent two different tissues, GO studies were performed separately to allow tissue- specific comparisons. GO terms were assigned to 7,304 midgut transcriptomic sequences (2,527 Biological Process, 585 Cellular Component and 1,169 Molecular Function; Figure 3 and Supplemental Table S2) and to 10,074 fat body transcriptomic sequences (2,827 Biological Process, 646 Cellular Component and 1,454 Molecular Function; Figure 4 and Supplemental Table S2).

### Metabolic pathways

We identified 115 KEGG metabolic pathways for the midgut and 122 for the fat body (Supplemental Table S3 and S4). Of the pathways identified, it was interesting to note that the beta-lactam resistance pathway was midgut-specific. However, at this point it is unclear as to what the functional role of this pathway may have specifically in the midgut. The specific pathways identified in the fat body were related to steroid biosynthesis, tetracycline biosynthesis, D-arginine and D-ornithine metabolism, ubiquinone and other terpenoid backbone biosynthesis. Besides these pathways, both the midgut and fat body databases also revealed transcripts for biosynthesis of alkaloids (derived from ornithine), lysine, nicotinic acid, histidine and purine.

### Protein domains

A Pfam domain search yielded 5,876 domains in 4,925 midgut transcripts and 8,852 domains in 7,205 fat body transcripts (Table 2). Among the identified Pfam domains, chitin binding Peritrophin-A (CBP-A) was found to be the highest in occurrence with 248 transcripts (4.2%) in the midgut and a mere 16 sequences (0.2%) in fat body. CBP-A domain is commonly found in proteins that constitute the peritrophic matrix (PM), a semipermA. planipennisle membrane that lines the gut of most insects [6], [32], [33]. PM assists in digestion and protecting the midgut epithelium from abrasion as well as from pathogens including bacteria, viruses and parasites [34], [35]. The presence of 0.2% fat body sequences with CBP-A domains is probably due to contamination during dissections and sample collection.

**Table 2:** Top Pfam domains identified in *Agrilus planipennis* sequences.

Pfam accession	Pfam domain description	Number of occurrence in midgut	Number of occurrence in fat body
PF01607.17	Chitin binding Peritrophin-A domain	248	16
PF00135.21	Carboxylesterase	58	27
PF00232.11	Glycosyl hydrolase family 1	48	14
PF00083.17	Sugar (and other) transporter	43	63
PF00069.18	Protein kinase domain	77	123
PF00400.25	WD domain, G-beta repeat	54	111
PF07714.10	Protein tyrosine kinase	52	91
PF00076.15	RNA recognition motifs	43	86
PF00067.15	Cytochrome P450	37	85
PF00096.19	Zinc finger, C_2_H_2_-type	36	82
PF00071.15	Ras family	66	73
PF00089.19	Trypsin	98	55
PF00153.20	Mitochondrial carrier protein	28	51

Several domains pertaining to detoxification enzymes were also identified in both the midgut and fat body transcripts of *A. planipennis*. First, compared to the fat body, the midgut showed a greater number of sequences matching domains of carboxylesterases (CEs). A similar high frequency of CEs was reported in the midgut of *C. tremulae* [30]. CEs are general detoxification enzymes involved in the resistance to carbamates, pyrethroids and organophosphate insecticides [36]. However, it is not clear what their role may be in the detoxification of plant allelochemicals [37]. Second, cytochrome P450 domains were found more frequently in fat body sequences than those of the midgut. These enzymes have been reported to detoxify a wide-range of xenobiotics including plant toxins and synthetic chemicals in an array of insect species [38].

WD-40 repeats were found in both midgut and fat body sequences. WD-repeat protein and its subunit domains were found significantly higher in fat body compared to the midgut (Table 2). WD-repeat proteins play an important role in RNA processing, signal transduction, cytoskeleton assembly, cell division and protein- protein interactions [29], [39]. In the fat body we identified the katanin domain, which is part of a microtubule severing protein (heterodimer of 60 and 80 kDa subunits) that targets the centrosome using WD-40 containing subunit [40]. Other identified domains that are potentially involved in signal transduction and cytoskeleton related functions were GTPase Ras families [41].

P-element wimpy testis-induced (PIWI) - interacting RNAs (piRNA) play an important role in gene regulation in addition to silencing retrotransposons and repetitive sequences during germline development [42], [43]. We identified some of the domains of proteins that are involved in the piRNA synthesis pathway. Interestingly, these domains were almost restricted to the fat body sequences of *A. planipennis* and included PIWI-like proteins and Tudor domain that are essential for piRNA production [42], [44]. The midgut sequences revealed only Tudor domains. piRNA differ from micro-RNAs (miRNAs) and small interfering RNAs (siRNAs) in several ways: 1) they interact only with PIWI and not with Argonaute; 2) piRNAs range from 24 to 31 nucleotide (nt) length compared to siRNAs and miRNAs, which are typically 21nt; and 3) piRNAs may positively regulate mRNA stability and translation whereas miRNAs and siRNAs have negative effect on transcriptional and translational process [45], [46]. To-date, the function of piRNA is unclear and deciphering the functional role of molecules involved in piRNA synthesis would contribute novel insights into gene regulation in insects.

Apart from the above-described domains we found a significant number of other domains pertaining to trypsins, glycosyl hydrolase family, sugar transporters, protein kinase domain, mitochondrial protein domains, C_2_H_2_ type zinc finger domains and RNA recognition motifs (Supplemental Table S5). The presence of a high number of trypsin and trypsin-like domains suggest that the gut of *A. planipennis* is predominantly composed of serine-proteases, which seems to be also one of the major classes of digestive proteases in the midgut of the beetle *C. tremulae* [30].

### Genes of interest

We are primarily interested in elucidating the functions of the genes involved in detoxification/antioxidant response (Table 3). Of particular interest are cytochrome P450s, which are known to be one of the most rapidly radiating groups of detoxifying enzymes in insects [47]. Cytochrome P450s metabolize toxic substrates including allelochemicals and insecticides to less harmful excretable forms via hydroxylation/oxidation [47], [48]. Cytochrome P450s have been classified into four large clades: CYP2, CYP3, CYP4, and Mitochondrial clade [47]. In this study, the predominant occurrence of *A. planipennis* cytochrome P450s was members of the CYP3 clade, which is in agreement with those reported in various insect orders including Diptera, Lepidoptera and Coleoptera [47]. The genome of the red flour beetle *T. castaneum* encodes about 147 cytochrome P450s of which 70 belong to the CYP3 clade [31]. To begin ascertaining putative function, we assessed the mRNA levels of candidate *A. planipennis* cytochrome P450 genes belonging to each of the four clades in different larval tissues (midgut, fat body and cuticle).

**Table 3:** Genes of interest in the midgut and fat body of *Agrilus planipennis* larvae.

Candidate genes	Number of occurrence in midgut	Number of occurrence in fat body
**Cytochrome P450**		
*CYP2 clade*	01	08
CYP2	—	01
CYP24	—	01
CYP49	—	01
CYP303	—	02
CYP306	01	03
*CYP3 clade*	37	45
CYP3	—	02
CYP6	26	37
CYP9	10	06
CYP28	01	—
*CYP4 clade*	03	16
CYP4	03	16
*Mitochondrial CYP clade*	04	06
CYP12	—	02
CYP301	03	01
CYP314	—	02
CYP315	01	01
**GST**	27	19
*Sigma*	08	01
*Omega*	01	02
*Theta*	17	15
*Microsomal*	01	01
Superoxide dismutase	05	07
Catalase	17	05
Carboxylesterases	16	05

**Figure 5:**
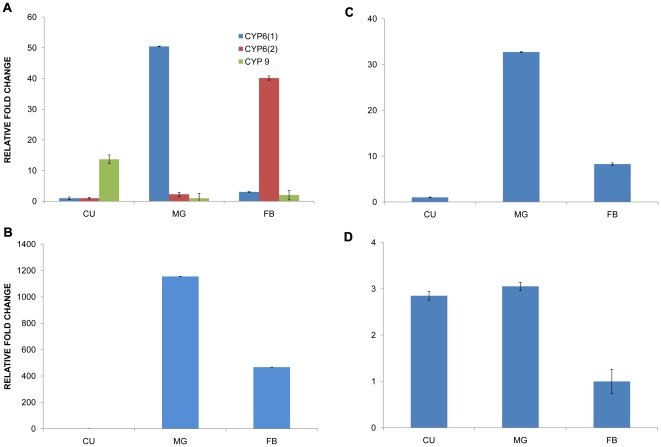
Relative mRNA levels of candidate cytochrome P450 genes in tissues of larval *Agrilus planipennis*. (A) Expression levels of CYP3 clade members including CYP6 and CYP9. CYP6(1) represents contig EABMG023904 and CYP6(2) represents contig EABMG001813, (B) Expression levels of a CYP4, (C) Expression levels of mitochondrial CYP clade CYP12 and (D) Expression levels of CYP306. Tissues assayed include cuticle (CU), midgut (MG) and fat body (FB). Standard error of the mean for two biological replicates (nested with two technical replicates) is represented by the error bars.

Members of the CYP3 clade (mainly CYP6 and CYP9) revealed distinct expression patterns. Of the two *A. planipennis* CYP6 genes assayed, EABMG023904 showed highest mRNA levels in the midgut while contig EABMG001813 showed higher transcript level in the fat body (Figure 5A). Similar mRNA levels of tissue-specific CYP6 genes have been observed in other insect species [49]–[51]. It is thought that the high expression levels of CYP6 genes were associated with metabolism of plant allelochemicals and insecticides [52]. However, qPCR analysis for a CYP9 member revealed highest mRNA levels in larval cuticle of *A. planipennis* (Figure 5A). CYP9 in *A. planipennis* larvae could be involved in xenobiotics metabolism as reported in other insect taxa [52], [53].

Although the CYP4 clade consists of numerous diverse genes, it seems to be the least studied group of cytochrome P450s in insects [48]. In this study, larval *A. planipennis* midgut showed the highest mRNA level for one of the CYP4 assayed (Figure 5B). Transcript levels in the fat body were significantly higher compared to levels in cuticle but significantly lower than the levels for the midgut. These observations are in agreement with CYP4 mode of action on dietary plant toxins reported in Lepidoptera (e.g. *Manduca sexta*; [54]) as well as against insecticides in Coleoptera (e.g. *Diabrotica virgifera virgifera* Leconte [55]). Apart from xenobiotic metabolism, members of CYP4 are also reported to have putative roles in ecdysteroid metabolism [56].

Of the CYP2 clade homologs identified in *A. planipennis* midgut, we assessed the expression of CYP306, which belongs to a set of cytochrome P450s called Halloween genes or otherwise known as the phantom gene, *phm* [57]. These genes are thought to be involved in ecdysone metabolism and therefore are important for insect growth and development. The CYP306 homolog of *A. planipennis* showed highest mRNA levels in the midgut and cuticle (Figure 5C). Similar tissue-specific profiles of CYP306 (*phm*) were observed in larva of *M. sexta* [57].

Mitochondrial P450s are very specific to animals and are not found in fungi and plants [48]. Insects have two types of mitochondrial P450, a conserved group involved in physiological functions and a diverse group known to participate in the detoxification of xenobiotics. Expression analysis of an *A. planipennis* CYP12 gene belonging to the mitochondrial clade revealed highest mRNA levels in the midgut compared to cuticle and fat body (Figure 5D), similarly to a study on resistance of *D. melanogaster* larvae to lufenuron, which was attributed to high expression of a CYP12 in the midgut and Malphigian tubules [58].

The above results suggest a potential involvement of cytochrome P450s in metabolism of host allelochemicals by *A. planipennis* larvae and perhaps in other physiological functions such as hormonal biosynthesis. However, additional experiments at the protein and transcript level (RNA interference) that elucidate gene function of these cytochrome P450s could provide insights into alternate control targets as in other insect systems [51].

### Molecular markers

We predicted 317 simple sequence repeats (SSRs or microsatellites) in *A. planipennis* midgut sequences and 571 SSRs in fat body sequences (Table 4). The majority of SSRs were either di-nucleotide (58 for midgut and 94 for fat body) or trinucleotide (223 for midgut and 428 for fat body) repeats; while 26 and 46 sequences of the midgut and fat body, respectively, were predicted to be tetra-nucleotide repeats and 10 and 3 sequences of the midgut and fat body, respectively, were predicted to be penta-nucleotide repeats. The characteristics of these SSRs including length, start, stop motif units and foot print are summarized in Supplemental Table S6. Interestingly, we also identified 137 putative single nucleotide polymorphisms (SNPs) in 45 sequences of the midgut and 347 SNPs in 143 sequences of the fat body (Table 5) (Supplemental Table S7). The exploitation of markers which are linked to coding regions would facilitate the selection of polymorphic markers for *A. planipennis* [59]. However, prior to making any inferences of the predicted molecular markers in *A. planipennis*, validation has to be performed to account for false positives.

**Table 4:** Putative microsatellite loci predicted in *Agrilus planipennis*.

Number of repeats	Di-nucleotide repeats		Tri-nucleotide repeats		Quad -nucleotide repeats		Penta -nucleotide repeats	
	MG*	FB	MG	FB	MG	FB	MG	FB
5			137	251	9	34	5	2
6			60	107	8	4	3	
7			18	45	3	5	2	1
8	19	38	1	16	4	1		
9	11	29	5	7	1			
10	9	12	1	2				
11	4	7			1			
12	6	4						
13	3							
14			1					
15	1	1				1		
16	1	1						
17	1							
18	1							
…								
21						1		
…								
23	1							
…								
27		2						
…								
46	1							
**Subtotal**	58	94	223	428	26	46	10	3

* MG  =  midgut; FB  =  fat body.

**Table 5:** Putative single nucleotide polymorphisms identified in *Agrilus planipennis*.

SNP type	MG*	FB
Transition		
A–G	52	124
C–T	37	111
Transversion		
A–C	9	20
A–T	17	41
C–G	8	30
G–T	14	21
Total	137	347

* MG  =  midgut; FB  =  fat body.

### Midgut microbiota

A small subset of the sequences (79 of 14680) showed closer similarity to microbial, rather than animal genes. They might thus represent low levels of microbial contamination in the dissection, or even horizontal transmission of genes from symbiotic microbes to the insect host (Supplemental Table S8). First, putative endo-glucanases were found that matched to *Streptomyces* species. Similar genera of microbiota in *A. planipennis* were identified by Vasanthakumar et al. [60]. In particular, they isolated *Streptomyces* sp. from midgut of larva; *Erwinia* sp. and *Burkholderia cepacia* from the guts of adult beetles. Second, putative endo-polygalacturonases were identified in the midgut of *A. planipennis*. Given that the diet of larval *A. planipennis* is primarily phloem-based, the recovered endo-glucanases and endo-polygalacturonases could facilitate in breakdown of plant cell walls [61] resulting in the release of nutrients required for larval growth and development.

A single bacterial sequence in the midgut transcriptome showed similarity with a *Wolbachia* sequence, an ubiquitous endosymbiont found across various insect taxa and nematodes [62]–[65]. *Wolbachia* is thought to interfere with insect reproduction through a process known as feminization, in which the sex ratios within a population become female-skewed [66], [67].

### Conclusions

This study represents the first report to elucidate tissue-specific gene expression in *A. planipennis*. Our analysis of *A. planipennis* midgut and fat body has revealed commonalities as well as potentially significant differences with other insect model systems and is beginning to provide insights into some of the fundamental physiological processes occurring in these tissues. Some noteworthy results of this study are 1) a significant number of cytochrome P450s that showed distinct tissue expression patterns; 2) a number of SNPs and microsatellite markers were predicted within the midgut and fat body databases, which upon validation could provide insights at functional polymorphism within and between tissues; and 3) some of the predicted microbial sequences encoding for enzymes involved in carbohydrate digestion and metabolism in the larval midgut of *A. planipennis* suggest the presence of putative endosymbionts. These traits along with the recovered sequences involved in piRNA synthesis provide substantial insights into the physiology-driven molecular mechanisms of *A. planipennis.*

## Materials and Methods

### Insect samples

*A. planipennis* larvae were collected from wood-lots consisting of green ash trees in Ottawa Lake, MI and Toledo, OH. Larvae were extracted by stripping off the bark of infested ash trees that were 10–25 cm in diameter at breast height. The ash trees sampled were alive but showed obvious symptoms of decline in response to *A. planipennis* colonization. The collected larvae were categorized according larval instars.

### Larval dissections and RNA isolation

About 50 late 3^rd^-instar larvae were dissected to extract midgut and fat body tissues. Dissections were performed as described by Vasanthakumar et al. [60]. The midgut tissue was dissected free of its contents and carefully rinsed in fresh PBS buffer prior to RNA isolation. Isolated larval midgut, cuticle and fatbody were placed in pre-chilled Trizol reagent in separate 1.5 ml eppendorf tubes. Following dissection, the tissues were stored at −80°C until RNA was isolated. The dissected tissues were homogenized separately in TRIzol® and total RNA was isolated following the manufacturer's protocol and stored at −80°C until use.

### cDNA library construction

The midgut and fat body RNA samples (10–20 µg) described above were used for constructing cDNA libraries. Prior to cDNA library construction, the total RNA was subjected to quality control using the Nanodrop 2000c (Thermo Scientific) and the RNA 6000 nanochip (Agilent Technologies) at the Purdue Genomics Core Facility (West Lafayette, IN). A SMART cDNA library construction kit (Clontech) was used to construct both libraries with modifications to suit the 454 GS FLX pyrosequencing. Instead of using the recommended MMLV reverse transcriptase, Super script II reverse transcriptase from Invitrogen was used for synthesizing the first-strand cDNA, and a modified PAGE-purified CDS II primer (5′ - TAG AGG CCG AGG CGG CCG ACA TGT TTT GTT TTT TTT TCT TTT TTT TTT VN - 3′) was used instead of the supplied one. The constructed cDNA libraries were shipped to the Purdue Genomics Core Facility for sequencing.

### 454 pyrosequencing

DNA bands of 500–800 bp were excised from the gel and purified. The isolated DNA was blunt ended, ligated to adapters and immobilized on Library Immobilization Beads. Blunt-end fragments were made with T4 polynucleotide kinase (PNK) and T4 DNA polymerase in a reaction that was held for 15 minutes at 12°C followed by 15 additional minutes at 25°C. The adaptors (Titanium A & B) used were provided with the 454 general library kit. The ligation reaction included ligase buffer, adaptors, and a ligase enzyme, which was incubated for 15 minutes at 25°C on a thermocycler. After the gaps were repaired, a single-stranded DNA library was isolated from the beads and quality controlled for the correct size using a LabChip 7500 machine. The concentration and the proper ligation of the adapters were examined using qPCR. One-quarter of a pico-titer plate was sequenced for each tissue sample (midgut and fat body) following manufacturer's protocol. The pyrosequencing was performed as per the Genome Sequencer FLX Operation (Roche).

### Bioinformatic data analysis

The transcriptome sequences of *A. planipennis* midgut and fat body were annotated by searching against the GenBank non-redundant database using BLASTx algorithms [68] The *A. planipennis* transcriptome sequences were compared to proteins of the fruit fly *Drosophila melanogaster*, the African malaria mosquito *Anopheles gambiae*, and the red flour beetle *Tribolium casteneum* using BLASTx algorithm [68]. Domains within the sequences were identified by searching against the Pfam database [69] using the HMMER v3 program [70]. We used Blast2GO [71], [72] to predict the functions of the sequences, assign Gene Ontology terms, and predict metabolic pathways in the KEGG database [73], [74]. Microsatellites/SSR markers were identified using Msatfinder version 2.0.9 program [75] and single nucleotide polymorphisms (SNPs) were predicted using the gsMapper program (Roche) with an arbitrary criterion of at least 4 reads supporting the consensus or variant.

### Quantitative real-time PCR

The concentration of total RNA samples was measured using NanoDrop 2000c Spectrophotometer from Thermo Scientific. The first strand cDNA was synthesized using Superscript IITM First Strand Synthesis kit for qRT-PCR from Invitrogen, following the manufacturer's protocol. For cDNA synthesis, 1 µl of oligo (dT)_12–18_ primer (500 µg/µl) and 1 µl of dNTPs mix (10 mM each) were added to 8 µl of total RNA sample (∼0.25 µg/µl). The samples were heated in a thermo cycler at 65°C for 4 min. Then, the following reagents were added on ice to each sample: 2 µl of 10×RT buffer, 4 µl of 25 mM MgCl_2_, 2 µl of 0.1 M DTT, 1 µl RNAse Out, 1 µl of SuperScript II reverse transcriptase and 1 µl of RNase H. The cycling parameters were 42°C for 90 min., 70°C for 15 min. and 37°C for 20 min. After cDNA synthesis, the concentrations of the first-strand samples were brought to 20 ng/µl, which were used as the template in qRT-PCR reactions. Cycling parameters included 95°C for 3 min and then, 40 cycles of 95°C for 10 s and 60°C for 30 s.

Quantification of candidate gene expression in tissues (midgut, fat body and cuticle) was based on the Relative Standard Curve method [2] using the threshold cycle (Ct) values. For all qRT-PCR analyses the mRNA coding for a ribosomal protein (RP7) was assessed and included as the internal control. Assessment of the mRNA encoding for ribosomal proteins has been demonstrated to be an appropriate internal control in other studies [76].

### Statistical analysis

Relative expression value (REV) for each gene was determined by dividing the quantities for the target samples by the quantities obtained for ribosomal protein. The REVs obtained for each gene in the tissues was analyzed by Analysis of Variance (ANOVA) (p = 0.05) using the PROC MIXED procedure of SAS (SAS Institute Inc. SAS.STAT User's Guide, Version 9.1). Two biological replicates nested with two technical replicates were included for the entire analysis. Biological replicates were included as a random effect in the model. Relative fold changes in the tissues were calculated by taking the sample (REV) that showed the least level of expression as 1X sample (calibrator,) [77]. The standard error represented the variance in the two biological replicates (2 technical replicates within each) included for this analysis.

### Data Deposition

The Roche 454 reads of *A. planipennis* midgut and fat body were submitted to NCBI Sequence Read Archive under the accession number of SRA012309.3.

## Supporting Information

Table S1 Genome comparisons of A. planipennis midgut and fat body sequences.
(6.16 MB XLS)

Table S2 Gene Ontology of A. planipennis midgut and fat body sequences.
(3.11 MB XLS)

Table S3 KEGG summary of A. planipennis midgut sequences.
(0.04 MB XLS)

Table S4 KEGG summary of A. planipennis fat body sequences.
(0.04 MB XLS)

Table S5 Pfam domain search of A. planipennis midgut and fat body sequences.
(2.59 MB XLS)

Table S6 Predicted microsatellite loci in A. planipennis midgut and fat body sequences.
(0.10 MB XLS)

Table S7 Predicted SNPs in A. planipennis midgut and fat body sequences.
(0.07 MB XLS)

Table S8 Midgut-specific microbiota sequences of A. planipennis.
(0.04 MB XLS)
